# Innovative employment of fistuloscope to cure complex ultra-low ileorectal anastomotic leakage

**DOI:** 10.1055/a-2860-0845

**Published:** 2026-05-12

**Authors:** Lei Wu, Zhitao Zhou, Jun Wang, Jianan Ren, Gefei Wang

**Affiliations:** 1 Research Institute of General SurgeryJinling Hospital, Affiliated Hospital of Medical School, Nanjing Medical UniversityNanjingChina; 2 Department of General SurgeryJinling Clinical Medical College, Nanjing University of Chinese MedicineNanjingChina; 3 Department of General Surgery 12581 Jinling Hospital, Affiliated Hospital of Medical School, Nanjing UniversityNanjingChina

Anastomotic leakage (AL) is a concerning yet unavoidable complication following colorectal surgery, associated with increased mortality and frequently requiring reoperation 1 2 . The management of AL, particularly ultra-low AL, presents a formidable clinical challenge. If not handled properly, the patient may face the risk of a permanent stoma or even death 3 . We report a case in which an ultra-slim cholangioscope was innovatively utilized as a fistuloscope for treating an ultra-low ileorectal AL.

**Fig. 1:**
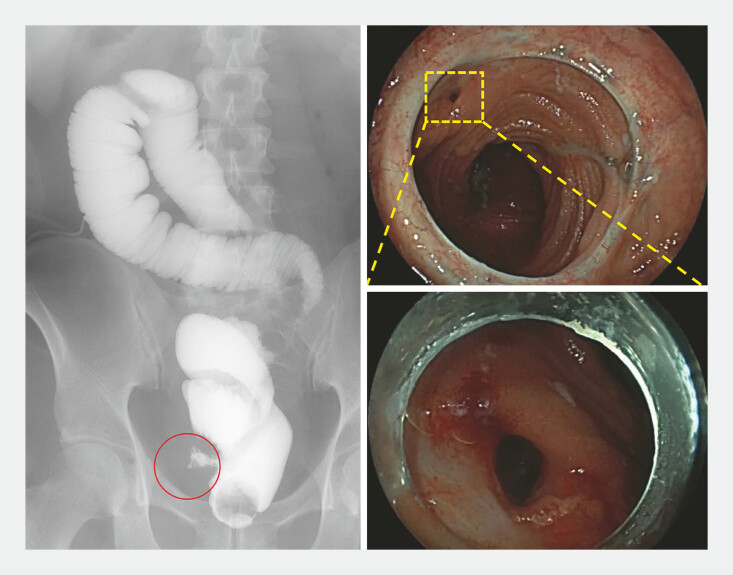
Colonoscopy and fistulography confirmed anastomotic leakage (red circle).

A 34-year-old man with a 10-year history of chronic constipation underwent laparoscopic total colectomy with ileorectal anastomosis. The anastomosis was constructed approximately 2 cm from the anal verge. On postoperative day 15, reoperation revealed a 0.4-cm leak on the posterior wall of the ileorectal anastomosis. Owing to severe adhesions and bleeding, the defect was not repaired, and an end ileostomy was created. Three months later, the patient presented with abdominal pain and fever. Colonoscopy and fistulography confirmed persistent AL ( Fig. 1 ). Endoscopic closure using an over-the-scope clip was attempted but failed due to extensive necrosis and the ultra-low location of the defect.

**Fig. 2:**
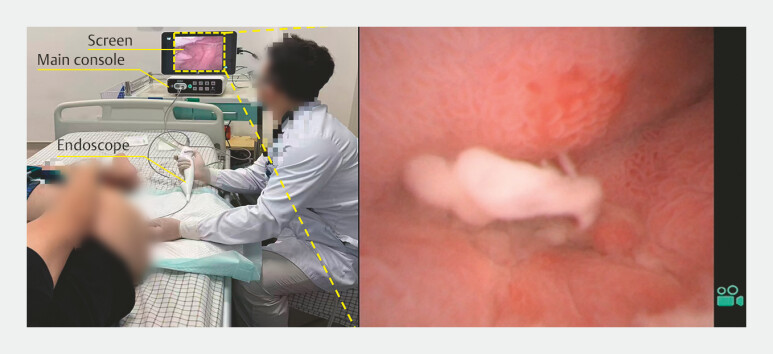
Appearance and operation of the fistuloscope.

**Fig. 3:**
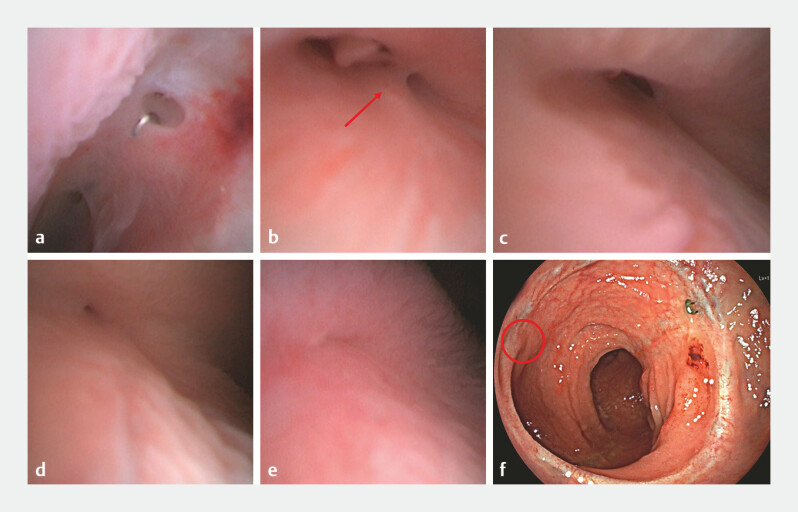
The process of fistula healing observed under fistuloscope: **a** fresh fistula, **b** gradual shrinkage of the fistula with bridging granulation tissue formation (red arrow), **c** significant reduction in fistula size, **d** near-complete healing of the fistula, and **e, f** complete healing of the fistula (the red circle indicates the healed site of the fistula).

Consequently, an ultra-slim cholangioscope (outer diameter 4 mm and working channel 2.8 mm) was used as a fistuloscope ( Fig. 2 ). Under direct visualization, the fistulous tract was irrigated and debrided, followed by closure with fibrin glue ( Video 1 ). Progressive reduction in fistula size was observed, with complete healing achieved ( Fig. 3 ). After approximately 1 month, a distal loopogram confirmed fistula closure, and stoma reversal was successfully performed. This technique enables the direct visualization of the fistulous tract, facilitates targeted debridement, and allows the real-time assessment of healing, thereby potentially expanding therapeutic options for ultra-low AL where conventional endoscopic approaches are limited.

Endoscopic management of an ultra-low ileorectal anastomotic leakage using an ultra-slim cholangioscope as a fistuloscope.Video 1

Endoscopy_UCTN_Code_TTT_1AO_2AI
